# Literature search on risk factors for sarcoma: PubMed and Google Scholar may be complementary sources

**DOI:** 10.1186/1756-0500-3-131

**Published:** 2010-05-10

**Authors:** Giuseppe Mastrangelo, Emanuela Fadda, Carlo R Rossi, Emanuele Zamprogno, Alessandra Buja, Luca Cegolon

**Affiliations:** 1 Department of Environmental Medicine and Public Health, Padua University, 35128 Padua, Italy; 2 Department of Oncological and Surgical Sciences, Padua University, 35128 Padua, Italy

## Abstract

**Background:**

Within the context of a European network dedicated to the study of sarcoma the relevant literature on sarcoma risk factors was collected by searching PubMed and Google Scholar, the two information storage and retrieval databases which can be accessed without charge. The present study aims to appraise the relative proficiency of PubMed and Google Scholar.

**Findings:**

Unlike PubMed, Google Scholar does not allow a choice between "Human" and "Animal" studies, nor between "Classical" and other types of studies. As a result, searches with Google Scholar produced high numbers of citations that have to be filtered. Google Scholar resulted in a higher sensitivity (proportion of relevant articles, meeting the search criteria), while PubMed in a higher specificity (proportion of lower quality articles not meeting the criteria, that are not retrieved). Concordance between Google Scholar and PubMed was as low as 8%.

**Conclusions:**

This study focused just on one topic. Although further studies are warranted, PM and GS appear to be complementary and their integration could greatly improve the search of references in medical research.

## Introduction

Within the context of a European network dedicated to the study of sarcoma, all incident cases have been collected for two years from the Pathology Departments of three European regions: Rhone-Alpes and Aquitaine in France and Veneto in Italy. All diagnoses of sarcoma were reviewed by the regional expert pathologist, and molecular subtypes were identified when possible. To find hypotheses that could be used to design a case-control study on risk factors for sarcoma, we collected the relevant literature by searching PubMed (PM) and Google Scholar (GS), the two information storage and retrieval databases which can be accessed without charge. The general characteristics of these search facilities have been recently presented [1-4]. The present study aims to appraise the relative proficiency of the two search engines.

## Methods

This is a study on concordance, sensitivity, specificity and accuracy of PM and GS in returning articles published between 2002 and 2009 on risk factors for sarcoma. The starting year was chosen because in late 2002 the World Health Organization published a new classification of sarcoma that gained widespread acceptance [5].

The papers shared by PM and GS - when adopting the same keywords and limits - were used to calculate a measure of concordance. Sensitivity, specificity and accuracy were assessed by using different searching strategies. The first was designed to be more sensitive and less specific: it was broader and could include both descriptive (ecological or clinical case series) and analytical (case control or cohort) epidemiological studies. The second strategy was instead more specific but less sensitive, focusing mainly on epidemiological analytical studies.

With two search engines (PM and GS) and two search strategies, there were four scenarios: GS1; GS2; PM1; PM2.

• GS1 started choosing all articles reporting the words "sarcoma", "incidence", and "case" (see string 1 in the box). Studies published before 2002 were excluded by using a special tool of GS. The list of papers was printed. In order to eliminate experimental animal studies and clinical studies on therapy and prognosis, retrieved articles presenting words such as "prognosis", "treat", "surg", "therapy", "efficacy", "survival", "chemotherapy", "mussel", "bivalve", "dog", "veterin", "cat", "feline", "bird", "avian", "fish", "mice", "rat", "mouse", "guinea", "rabbit", "ocean", "Kaposi's", "osteosarcoma", "Rous" (see string 2 in the box) were removed, but only after reading the title. Among the remaining articles, those not reporting "sarcoma" in the title or abstract, or being citations of books, abstract conferences, letters to the editor, or editorials were discarded. Interface was English, but pages written in any tongue were searched. Papers in language other than English were eventually ruled out.

• GS2 used the same search method as GS1, except that the searched terms were "sarcoma", "incidence", "case", and "risk" (see strings 3 and 4 in Additional file 1: Box).

• PM1 started by selecting articles reporting the words "sarcoma", "incidence", and "case" (see string 5 in the box). Furthermore, the following "Limits" were chosen: "Humans"; "English language"; "Classical article" as type of article; and "January 1^st ^2002 - April 22^th ^2009 as specific date range. After printing the list, the papers were scrutinized for the words "prognosis", "treat", "surg", "therapy", "efficacy", "survival", "chemotherapy", "Kaposi's", "osteosarcoma", "Rous" (see string 6 in box). Again, the same "Limits" as above were applied. The remaining articles were inspected for the presence of the word "sarcoma" in either the title or the abstract and those not reporting "sarcoma" in the title or the abstract were discarded. Clinical studies on diagnosis/therapy/prognosis were eventually discarded.

• PM2 used the same search method as in PM1, except that the searched terms were "sarcoma", "incidence", "case", and "risk" (see strings 7 and 8 in Additional file 1: Box).

After abstract selections were agreed upon, the filtered papers were evaluated by two independent reviewers in order to establish whether the risk factors for sarcoma were investigated or not. In the latter instance papers were mainly case reports or described new diagnostic devices (e.g. molecular biology or imaging techniques). Disagreements between reviewers concerning classification of articles were resolved by discussion and input from a third reviewer. Finally, the whole number of 168 (= 63+42+46+17) studies collected was reduced to 111 (common list) by excluding those shared by the parent lists GS1, GS2, PM1, and PM2.

### Statistical analysis

The common list was broken down into a series of two-by-two tables, in which columns were headed "Yes RF" and "No RF" - depending on whether the risk factors (RF) for sarcoma were or were not investigated - and rows were GS1 (or, in turn, GS2, PM1, PM2) and the remaining sources altogether. In such tables we calculated sensitivity, specificity, precision and accuracy.

The sensitivity for a given strategy is defined as the proportion of articles retrieved that are scientifically sound and clinically relevant (high quality articles); specificity is the proportion of lower quality articles (did not meet criteria) that are not retrieved; precision is the proportion of retrieved articles that meet criteria (equivalent to positive predictive value in diagnostic test terminology); and accuracy is the proportion of all articles that are correctly dealt with by the strategy (articles that met criteria and were retrieved plus articles that did not meet criteria and were not retrieved divided by all articles in the database) [6].

The common list was also broken down to show papers shared by pairs of bibliographic sources (GS 1 and GS2; PM1 and PM2; GS and PM) that used the same key words and limits. The agreement between pairs was calculated using the Dunn's method [7].

## Results

**Figure 1:**
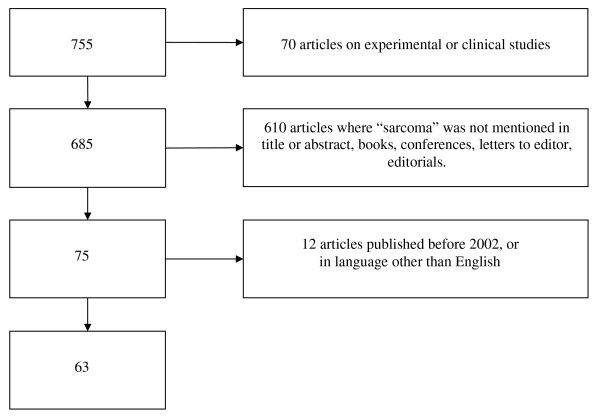
Flow diagram of articles retrieved by GS1 and numbers of subsequent exclusions.

Figure 1 shows a flow diagram of the exclusion criteria applied to the articles retrieved by GS1. Out of the initial 755 items returned by GS1, approximately 90% were discarded for the following differing reasons: 9% (= 70/755) were excluded because they were animal experimental studies or human clinical ones; 80% (= 610/755) were excluded because the word "sarcoma" was mentioned in the Introduction, Discussion or References (but not in the Title or Abstract) or because citations concerned publications such as editorials, books, letters to editors, conferences, posters, etc.; 2% (= 12/755) because the language was not English. The number of the remaining relevant papers was 63.

**Figure 2:**
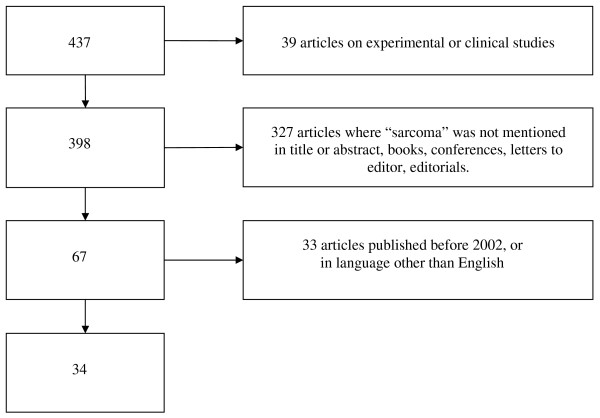
Flow diagram of articles retrieved by GS2 and numbers of subsequent exclusions.

Figure 2 shows a flow diagram of the exclusion criteria applied to the articles retrieved by GS2. Out of the initial 437 items returned by GS2, approximately 91% were discarded for the following reasons: 9% (= 39/437) were excluded because they were animal experimental studies or human clinical ones; 75% (= 327/437) were rejected because the word "sarcoma" was mentioned in the Introduction, Discussion or References (but not in the Title or Abstract) or because citations concerned publications such as editorials, books, letters to editors, conferences, posters, etc.; 8% (= 33/437) because the language was not English. The number of the remaining relevant papers was 34.

**Figure 3:**
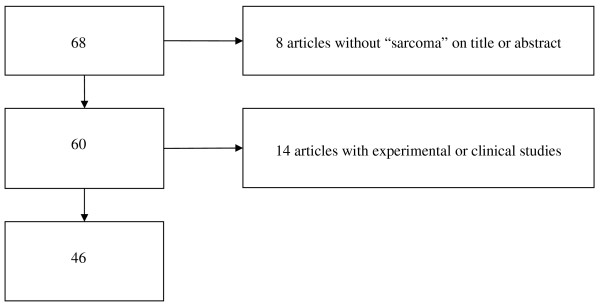
Flow diagram of articles retrieved by PM1 and numbers of subsequent exclusions.

Figure 3 shows a flow diagram of the exclusion criteria applied to the articles retrieved by PM1: the initial number returned by PM1 (68) was remarkably smaller than that provided by GS1 (755) and GS2 (437). 12% (= 8/68) of articles were excluded because they did not report "sarcoma" in the Title or Abstract; 21% (= 14/68) were ruled out as being clinical studies. The final number of relevant articles was 46, 68% (= 46/68) of the initial number.

**Figure 4:**
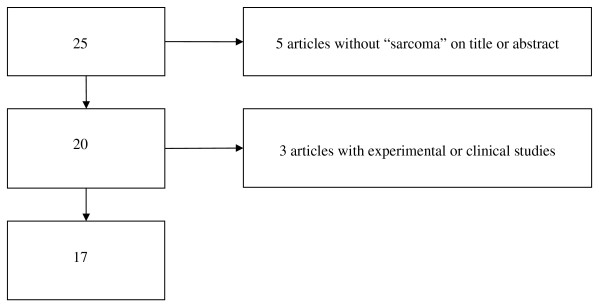
Flow diagram of articles retrieved by PM2 and numbers of subsequent exclusions.

Figure 4 shows a flow diagram of the exclusion criteria applied to the articles retrieved by PM2: the initial number returned by PM2 (25) was remarkably smaller than that provided by GS1 (755) and GS2 (437). 20% (= 5/25) of articles were excluded because they did not report "sarcoma" in the Title or Abstract; 12% (= 3/25) were ruled out as being clinical studies. The final number of relevant articles was 17, 68% (= 17/25) of the initial number.

The Additional File 1: Table S1 shows that, as expected, GS2 and PM2 were more specific, precise and accurate strategies than GS1 and PM1. Furthermore GS was more sensitive yet less specific as compared to PM. The two search engines showed similar precision and accuracy.

Additional file 1: Table S2 shows that the agreement between pairs of bibliographic sources was poor, especially in the comparison between GS and PM, where this concordance was as low as 8%.

## Discussion

A possible limitation of this study is the decision to exclude articles written in language other than English, thus ruling out items written in different language that could provide relevant information for systematic reviews of the literature. Papers written in languages other than English were discarded because they could be published in journals not indexed in PM; nevertheless these items could be found in GS as its inclusion criteria are obscure.

Another possible limitation of this study is that it focused just on one topic. However, our methodology of checking the retrieved articles - one by one - is hardly applicable to other medical research fields where GS could browse and return up to several hundred thousands of papers. Despite the difficulty in predicting the external validity of our results, we believe that our findings might be reasonably applied to other biomedical subjects.

In our study GS returned more results than PM, but most citations were unnecessary. Roughly 80% of citations of GS rely on items which can be any sort of publication (books, conferences acts, posters, letters to editor, editorials, etc.), including scientific articles where the key word is cited by chance in the introduction, discussion, or references. Indeed, unlike PM, GS does not allow a clear selection between "Human" and "Animal" studies. Punctuation characters in titles produce incorrect search results, and authors are assigned to the wrong papers, hence leading to erroneous additional search results. Some of these are even provided with no comprehensible reason. This is perhaps the primary reason why Google Scholar returns a greater number of results than PM and why there are many GS results which are off topic or "false hits". These facts produce a huge numbers of citations that have to be examined one by one in order to exclude those of no interest.

The present findings were confirmed by Falagas [1] - who found that GS could help in retrieving even the most obscure information, but offers results of inconsistent accuracy - and Shultz [3], reporting that GS provided more results than PM in 8 out of 10 test searches. Furthermore according to Freeman [4], PM searches yielded fewer total citations than GS results, but PM appeared to be more specific than GS for locating relevant primary literature articles for drug-related questions. In spite of the above, Google is the search engine of choice for more than half of all Web queries [8-10].

We found that GS searches yield more free, full-text journal articles as compared to PM. We investigated whether GS citations not provided by PM could be found in Medline and discovered that most articles located in GS were accessible from PM by searching for "Author".

Therefore the two search engines seem complementary. Since they are both free for all users, their integration could greatly improve the search of references in medical research.

## Conclusions

Citations from GS were more sensitive, while citations from PM were more specific. Interestingly, most GS citations not provided by PM were found in the Medline database by searching for "Author". This study focused just on one topic. Although further studies are warranted, PM and GS appear to be complementary and their integration could greatly improve the search of references in medical research.

## Competing interests

The authors declare that they have no competing interests.

## Authors' contributions

GM conceived the study, and participated in its design and coordination, LC contributed with the statistical analysis and the drafting of the paper, CRC is the guarantor, EF participated in the literature search, EZ and AB provided technical advice. All authors read and approved the final manuscript

## Supplementary Material

Additional file 1 **Box; Table S1; Table S2 (a, b and c)**. - Box: search strings employed in the different searching strategies: Google Scholar 1 (GS1); Google Scholar 2 (GS2); PubMed 1(PM1); PubMed 2 (PM2). - Table S1: Sensitivity, specificity and precision of the different sources of citations/search strategies; - Table S2: Two by two table displaying the papers shared by pairs of bibliographic sources. Click here for file
