# Supplementary material for: Literature search on risk factors for sarcoma: PubMed and Google Scholar may be complementary sources
Source: BMC Res Notes. 2010 May 10;3:131. doi: 10.1186/1756-0500-3-131 (PMC2874568; doi:10.1186/1756-0500-3-131)
Supplement: Additional file 1 — Box; Table S1; Table S2 (a, b and c). - Box: search strings employed in the different searching strategies: Google Scholar 1 (GS1); Google Scholar 2 (GS2); PubMed 1(PM1); PubMed 2 (PM2). - Table S1: Sensitivity, specificity and precision of the different sources of citations/search strategies; - Table S2: Two by two table displaying the papers shared by pairs of bibliographic sources. [file 1756-0500-3-131-S1.DOC]

| Box. Search strings in the different searching strategies (see text): Google Scholar 1 (GS1); Google Scholar 2 (GS2); PubMed 1(PM1); PubMed 2 (PM2). | | |
| --- | --- | --- |
| STRATEGY | | SEARCH STRINGS (AND SEARCH LIMITS FOR PUBMED) |
| GS1 | String 1 | “sarcoma incidence case”. |
|  | String 2 | "sarcoma incidence case –prognosis –treat -surg –therapy –efficacy –survival –chemotherapy –mussel –bivalve –dog –veterin –-cat –feline –bird –avian –fish –mice –rat –mouse –guinea –rabbit –ocean –Kaposi’s –Rous – osteosarcoma” ù |
| GS2 | String 3 | “sarcoma incidence case risk” |
|  | String 4 | “sarcoma incidence case risk –prognosis –treat -surg –therapy –efficacy –survival –chemotherapy –mussel –bivalve –dog –veterin –-cat –feline –bird –avian –fish –mice –rat –mouse –guinea –rabbit –ocean –Kaposi’s –Rous – osteosarcoma” |
| PM1 | String 5 | “sarcoma AND incidence AND case”. The following “Limits” were chosen: Humans; English language; Classical article as type of article; and January 1^st^ 2002 – April 22^th^ 2009 as specific date range. |
|  | String 6 | “sarcoma AND incidence AND case NOT(surg* prognosis treat therap* efficacy survival chemotherapy Kaposi Rous)”. The following “Limits” were chosen: Humans; English language; Classical article as type of article; and January 1^st^ 2002 – April 22^th^ 2009 as specific date range. |
| PM2 | String 7 | “sarcoma AND incidence AND case AND risk”. The following “Limits” were chosen: Humans; English language; Classical article as type of article; and January 1^st^ 2002 – April 22^th^ 2009 as specific date range. |
|  | String 8 | “sarcoma AND incidence AND case AND risk NOT(surg* prognosis treat therap* efficacy survival chemotherapy Kaposi Rous)”. The following “Limits” were chosen: Humans; English language; Classical article as type of article; and January 1^st^ 2002 – April 22^th^ 2009 as specific date range. |

| **Table 1. Sensitivity, specificity and precision of the different sources of citations/search strategies.** | | | | | | | | | |  |
| --- | --- | --- | --- | --- | --- | --- | --- | --- | --- | --- |
|  |  |  | |  |  |  |  | |  | |
|  |  | Common list | |  |  |  | Common list | |  | |
|  |  | Yes RF | No RF |  |  |  | Yes RF | No RF |  | |
|  |  |  |  |  |  |  |  |  |  | |
| Parent list | GS1 | 25 | 38 | Sensitivity = 25/43 = 58% | Parent list | GS2 | 26 | 16 | Sensitivity = 26/43 = 60% | |
|  | Other | 18 | 30 | Specificity = 30/68 = 44% |  | Other | 17 | 52 | Specificity = 52/68 = 76% | |
| Total |  | 43 | 68 | Precision = 25/(25+38) = 40% | Total |  | 43 | 68 | Precision = 26/(26+16) = 62% | |
|  |  |  |  | Accuracy = 55/111= 50% |  |  |  |  | Accuracy = 78/111= 70% | |
|  |  |  |  |  |  |  |  |  |  | |
|  |  |  |  |  |  |  |  |  |  | |
|  |  |  |  |  |  |  |  |  |  | |
|  |  | Common list | |  |  |  | Common list | |  | |
|  |  | Yes RF | No RF |  |  |  | Yes RF | No RF |  | |
|  |  |  |  |  |  |  |  |  |  | |
| Parent list | PM1 | 19 | 27 | Sensitivity = 19/43 = 44% | Parent list | PM2 | 15 | 2 | Sensitivity = 15/43 = 35% | |
|  | Other | 24 | 41 | Specificity = 41/68 = 60% |  | Other | 28 | 66 | Specificity = 66/68 = 97% | |
| Total |  | 43 | 68 | Precision = 19/(19+27) = 41% | Total |  | 43 | 68 | Precision = 15/(15+2) = 88% | |
|  |  |  |  | Accuracy = 60/111=54% |  |  |  |  | Accuracy = 81/111=73% | |
|  |  |  |  |  |  |  |  |  |  | |
|  |  |  |  |  |  |  |  |  |  | |
|  |  |  |  |  |  |  |  |  |  | |
|  |  | Common list | |  |  |  | Common list | |  | |
|  |  | Yes RF | No RF |  |  |  | Yes RF | No RF |  | |
|  |  |  |  |  |  |  |  |  |  | |
| Parent list | Scholar | 30 | 44 | Sensitivity = 30/43 = 70% | Parent list | PubMed | 19 | 27 | Sensitivity = 19/43 = 44% | |
|  | Other | 13 | 24 | Specificity = 24/68 = 35% |  | Other | 24 | 41 | Specificity = 41/68 = 60% | |
| Total |  | 43 | 68 | Precision = 30/(30+44) = 41% | Total |  | 43 | 68 | Precision = 19/(19+27) = 41% | |
|  |  |  |  | Accuracy = 54/111= 49% |  |  |  |  | Accuracy = 60/111= 54% | |
| Yes RF = sarcoma risk factors were investigated  NO RF = sarcoma risk factors were not investigated | | | | | | | | | |  |
| GS1, GS2 = Strategy Google Scholar 1 and 2 (see text) | | | | | | | | | |  |
| PM1, PM2 = Strategy PubMed 1 and 2 (see text) | | | | | | | | | |  |
| Scholar = GS1 and GS2; PubMed = PM1 and PM2 | | | | | | | | | |  |

| Table 2a. Papers shared by PubMed 2 and PubMed 1, using the same key words and limits. | | | | |
| --- | --- | --- | --- | --- |
|  | | Pub Med 2 | |  |
|  | | Yes | No | Total |
| PubMed 1 | Yes | 17 | 29 | 46 |
|  | No | 0 |  |  |
|  | Total | 17 |  |  |
| Concordance measure [Dunn, 1992]: 17/(17+0+29) = 37% | | | | |

| Table 2b. Papers shared by Scholar 2 and Scholar 1, using the same key words and limits. | | | | |
| --- | --- | --- | --- | --- |
|  | | Scholar 2 | |  |
|  | | Yes | No | Total |
| Scholar 1 | Yes | 31 | 32 | 63 |
|  | No | 11 |  |  |
|  | Total | 42 |  |  |
| Concordance measure [Dunn, 1992]: 31/(31+32+11) = 42% | | | | |

| Table 2c. Papers shared by Scholar and PubMed, using the same key words and limits. | | | | |
| --- | --- | --- | --- | --- |
|  | | PubMed | |  |
|  | | Yes | No | Total |
| Scholar | Yes | 9 | 65 | 74 |
|  | No | 37 |  |  |
|  | Total | 46 |  |  |
| Concordance measure [Dunn, 1992]: 9/(9+65+37) = 8% | | | | |
